# Individual and community-level determinants of knowledge about fertile periods among adolescent girls and young women (10–24 years) in Sub-Saharan Africa: A multilevel mixed effect analysis of a recent demographic and health survey

**DOI:** 10.1016/j.heliyon.2024.e26756

**Published:** 2024-02-24

**Authors:** Alebachew Ferede Zegeye, Tadesse Tarik Tamir, Enyew Getaneh Mekonen, Belayneh Shetie Workneh, Wubshet Debebe Negash, Chilot Kassa Mekonnen

**Affiliations:** a Department of Medical Nursing, School of Nursing, College of Medicine and Health Sciences, University of Gondar, Gondar, Ethiopia; b Department of Pediatrics and Child Health Nursing, School of Nursing, College of Medicine and Health Sciences, University of Gondar, Gondar, Ethiopia; c Department of Surgical Nursing, School of Nursing, College of Medicine and Health Sciences, University of Gondar, Gondar, Ethiopia; d Department of Emergency and Critical Care Nursing, School of Nursing, College of Medicine and Health Sciences, University of Gondar, Gondar, Ethiopia; e Department of Health Systems and Policy, Institute of Public Health, College of Medicine and Health Sciences, University of Gondar, Gondar, Ethiopia

**Keywords:** Adolescent girls, Determinants, Fertile period, Knowledge, Sub-Saharan Africa, Yong women

## Abstract

**Background:**

Identifying fertility periods accurately will protect teenage girls and young women from unintended pregnancies and related complications. However, in Sub-Saharan Africa, knowledge of the fertile period among adolescent girls and young women is not well studied. Thus, the purpose of this study was to assess adolescents' and young women's knowledge regarding fertility periods and its determinants in Sub-Saharan Africa.

**Methods:**

The most recent demographic and health surveys' data were used. The study had 140,064 participants in all. The data was analyzed using STATA/SE version 14. Using a multilevel logistic regression model, factors associated with knowledge of fertile periods have been determined. P-values <0.05 showed the significance of the factors associated with the outcome variable. The data were interpreted using the adjusted odds ratio and confidence interval. The best-fit model was determined to be the one with the highest logliklihood ratio and the lowest deviance.

**Results:**

One in five (20%) women between the ages of 10 and 24 had accurate knowledge about the fertile period. Age (AOR = 1.44, 95% CI: 1.40, 1.49), educational level (AOR = 1.68, 95% CI: 1.60, 1.77), knowledge of family planning (AOR = 1.33, 95% CI: 1.27, 1.39), distance (AOR = 2.31, 95% CI: 2.02, 2.98), residence (AOR = 1.06, 95% CI: 1.02, 1.10), and low community poverty (AOR = 3.06, 95% CI: 3.01, 3.12) had higher odds of knowledge about fertile period.

**Conclusion:**

This research finds that adolescents and young women in sub-Saharan Africa have low knowledge of the reproductive period. It was discovered that factors at the individual and communal levels influence women's knowledge of the fertile period. While developing policies and strategies, the health ministries of Sub-Saharan African countries had to take into consideration input from women whose experiences indicated that obtaining reproductive health services was hindered by distance.

## 1 Background

The World Health Organization (WHO) defined adolescent girls and young women as females between the ages of 15 and 24, who account for about 20% of the global population. Teenage girls and young women are at risk for serious health issues because they are going through a major physiological, psychological, and social transition from childhood to adulthood [1,2]. The fertile period is the time during which a woman can conceive. One of the natural family planning techniques used to delay pregnancy is awareness of the fertile period. The natural techniques that are useful for identifying the fertile period are the cervical mucus method, the symptom thermal approach, and the basal body temperature method [3,4]. The accurate identification of the fertile period during the menstrual cycle is crucial for women who choose not to utilize mechanical, hormonal, or surgical methods of contraception in order to prevent unwanted pregnancies [5].

Each year, more than 16 million young women give birth in the world, with more than 50% occurring in Sub-Saharan Africa. Early pregnancies, especially in Sub-Saharan Africa, have both direct and indirect detrimental health and social effects on adolescent girls and young women, their families, and communities. Early pregnancy has direct negative impacts on an individual's health and social life, such as school dropout, extended labor, early birth, stillbirths, neonatal deaths, and maternal and perinatal mortality. Indirect detrimental health and social effects of early pregnancies include growing up in individual and community poverty, low school performance of the child, inadequate support, absence of a father figure in the family, and an unstable family structure, and poor physical and mental health development of the child [[6], [7], [8]]. Unsafe abortions account for almost half of all maternal deaths, which tend to be brought on by unintended pregnancies. One method of preventing unwanted pregnancies is to be aware of the fertile period [9].

Accurate knowledge of the fertile window will save young women and adolescent girls from unplanned and unwanted pregnancies. Those who do not utilize contraception and are not aware of when they are fertile are also at a higher chance of becoming pregnant unexpectedly. Understanding the fertile period is therefore one of the most significant contributing indications to the reduction in high fertility, which has negative implications for economic and social development [10,11]. Young women would be more likely to understand the risks of pregnancy, plan their pregnancies effectively, and detect their pregnancies early if they had access to adequate knowledge about fertile periods [12]. Therefore, having sufficient knowledge of the period of ovulation or being aware of fertility may help lower the incidence of unintended pregnancies, particularly in Sub-Saharan African (SSA) countries where adolescent and young women's access to reproductive health services is poor [[13], [14], [15], [16]].

Numerous studies conducted globally have demonstrated an association between women's age, educational attainment, marital status, place of residence, and media exposure and their knowledge of the reproductive periods of young women [[17], [18], [19]]. In economically disadvantaged countries, including sub-Saharan Africa, where a significant portion of maternal morbidity and mortality are overwhelmingly attributable to unintended pregnancies, community-level determinants of knowledge of the fertile period have not been well studied, despite some studies conducted globally showing an association between knowledge of the fertile period and some individual-level variables.

Hence, this study investigated teenage girls' and young women's knowledge about the fertile periods in sub-Saharan Africa and its determinants using data from the most recent Demographic and Health Survey (DHS). In order to mitigate the incidence of unwanted pregnancies in developing countries, particularly those in sub-Saharan Africa, the study's findings also support health planners, policymakers, and medical professionals who would like to increase sexual education and preventive interventions for young women and adolescents who are deemed to be at-risk.

## 2 Methods and materials

### 2.1 Study setting

The region of Africa south of the Sahara is known as the sub-Saharan, and it is made up of four huge and diversified regions: Eastern Africa, Central Africa, Western Africa, and Southern Africa. Collectively, they include an area of 9.4 million square miles and are inhabited by 1.3 billion people, of which half will be under 25 by 2050 [20,21]. Twenty-three sub-Saharan African countries, including Angola, Benin, Burundi, Cameron, Ethiopia, Ghana, Gambia, Guinea, Kenya, Liberia, Lesotho, Mali, Malawi, Nigeria, Rwanda, Serra Leone, Senegal, Chad, Tanzania, Uganda, South Africa, Zambia, and Zimbabwe, provided data for this study.

### 2.2 Study design and period

A cross-sectional study with a community focus was carried out. A multilevel mixed effect analysis has been conducted using data from 23 sub-Saharan African countries that were surveyed between 2014 and 2020. As a component of the global Demographic and Health Survey, the Demographic and Health Survey (DHS) is a five-year national study that employs organized, pretested, and tested tools. To get a representative sample of recent Demographic and Health Survey data from each country in sub-Saharan Africa, six years of DHS data (starting in 2014) have been appended. Large sample sizes are included in these population-based, nationally representative surveys of all countries.

### 2.3 Population and eligibility criteria

The source population consisted of young women and adolescent girls in Sub-Saharan African countries, ages 10 to 24. All of the adolescent girls and young women who live in the selected enumeration areas that were included in the analysis made up the study population.

### 2.4 Data source and sampling procedure

In order to assess adolescent girls and young women's knowledge of the fertile period and its determinates, the DHS survey data from sub-Saharan countries was incorporated jointly. Different datasets, such as those related to nutrition, reproductive health, fertility, and maternity and child health, as well as adult self-reported health behavior, are included in each country's survey. Using a stratified two-stage cluster design, the Demographic and Health Survey first creates the enumeration regions and then generates a sample of households from each enumeration area in the second stage. The outcome variable (knowledge of fertile period) has been generated by recoding the variable knowledge of fertile period (v217) from the individual record (IR) data set. The association of knowledge of the fertile period and its determinants was assessed using a binary logistic regression model.

To make the analysis survey-specific, we used the weighting variable (v005) as a relative weight normalized. For the pooled data, we were able to denormalize the variable by dividing each adolescent girls and young woman's standard weight by the sampling percentage for each country. The adjusted weight of adolescent girls and young women is equal to V005 × (the total number of women in the country between the ages of 10 and 24 at the time of the survey)/(the number of women in the country between the ages of 10 and 24 who were interviewed for the survey). Lastly, the study's total weighted sample consisted of 140,064 adolescent girls and young women (Table 1).

**Table 1:** Sample size for knowledge of the fertile period and its determinants among adolescent girls and young women in Sub-Saharan African, DHS 2014–2020.

Country	Survey year	Sample (weighted) (n)	Sample (weighted) (%)
Angola	2015	6423	4.59
Benin	2017/18	6251	4.46
Burundi	2016/17	7218	5.15
Cameron	2018	5812	4.15
Ethiopia	2016	6401	4.57
Ghana	2015	3327	2.38
Gambia	2019	4769	3.4
Guinea	2018	4267	3.05
Kenya	2014	11,483	8.2
Liberia	2019/20	3124	2.23
Lesotho	2014	2842	2.03
Mali	2018	4116	2.94
Malawi	2015	10,367	7.4
Nigeria	2018	15,267	10.9
Rwanda	2019/20	5732	4.09
Sera Leone	2019	6062	4.33
Senegal	2019	3612	2.58
Chad	2014/15	6884	4.91
Tanzania	2015	5399	3.85
Uganda	2016	8058	5.75
South Africa	2016	2913	2.08
Zambia	2018	5799	4.14
Zimbabwe	2015	3938	2.81
Total sample size		140,064	100

## 3 Study variables

**Dependent variables:** The independent variable of this study was the knowledge of fertile periods, which was classified into two categories: accurate knowledge and inaccurate knowledge. The knowledge of the ovulation period was assessed by asking respondents a single question: "When do you think the ovulation period of a woman is?" Respondents who stated that the fertile period is in the middle of the menstrual cycle responded to the knowledge of fertile period (v217) variable from the individual record (IR) data set were categorized as having accurate knowledge of fertile period, while respondents who answered the question as "during her period," "after her period ended," "before her period begins," "at any time," and "I don't know" were categorized as having inaccurate knowledge about fertile period [22,23]. Independent variables (Table 2)

**Table 2:** Summary of the dependent and independent variables for adolescents and young women in Sub-Saharan Africa on the knowledge of the fertile period and its determinants, DHS 2014–2020.

Study variables	
Dependent variable	Independent variables
✓ knowledge of fertile periods (accurate, inaccurate)	**Individual-level independent factors** ✓age 15–19, 20–24) ✓residence (rural or urban) ✓Religion (Catholic. Muslim, Orthodox, Protestant, Others) ✓occupation (not working, employed, Sales, agriculture, others) ✓educational status (not educated, primary, secondary, and higher) ✓marital status (never married, married now, previously/ever married) ✓media exposure (yes, no) ✓internet utilization (yes, no) ✓household wealth index (poor, middle, rich) ✓heard about family planning (yes, no)
	**Community-level factors** ✓Residence (rural or urban) ✓Community level illiteracy (Low, High) ✓Community level poverty (Low, High) ✓Community level media exposure (Low, High) ✓Country category (Southern, Eastern, West, and Central SSA)

### 3.1 Statistical analysis and data processing

STATA/SE version 14 statistical software was used to extract, clean, record, and analyze the data from recent DHS data sets. The models were compared using the deviance and log-likelihood tests; the model with the highest log-likelihood ratio and the lowest deviance was found to be the best-fitting one. Additionally, the variance inflation factor (VIF) was used to test for multicollinearity. The results show that there was no significant multicollinearity across the independent variables, with a mean VIF of 1.53.

The hierarchical nature of DHS data violated the assumptions of independent observations and equal variance across clusters, making the standard logistic regression model inapplicable. This suggests that an advanced model is needed to account for between-cluster variations. In considering this, multilevel mixed-effects logistic regression was employed to identify factors associated to the knowledge of the period of fertility. Multilevel mixed effect logistic regression uses four models: model I, which only includes variables at the individual level; model II, which only includes variables at the community level; and model III, which includes variables at both the individual and community levels. The model without independent variables (the null model) was used to check the variability of knowledge of fertile periods across the cluster. Model I evaluated the association between the outcome variable and the individual-level factors, while Model II evaluated the association between the community-level factors and the outcome variable. In the final model (Model III), the association of both individual and community-level variables was fitted simultaneously with the outcome variable (knowledge of fertile period).

### 3.2 Random effects (measures of variation)

The intra-class correlation coefficient (ICC), proportional change in variance (PCV), and median odds ratio (MOR) were used to assess random effects or measures of variation of the outcome variables (knowledge of fertile period). To quantify the difference across clusters, the proportional change in variance (PCV) and intra-class correlation coefficient (ICC) have been determined. Taking clusters as a random variable, the ICC reveals the variation of knowledge on fertile period between clusters is computed as: $ICC=\frac{VC}{VC+3.29}\times 100\%$. When two clusters are randomly selected, using clusters as a random variable, the MOR is the median value of the odds ratio between the area of the highest and the area of the lowest for knowledge of fertile periods; MOR = *e* ^0.95√VC^

Furthermore, the PCV, which is calculated as Vnull = variance of the null model and VC = cluster level variance, illustrates the variation in knowledge of the fertile period explained by determinants [24]. Using the fixed effects technique, the probability of knowledge about fertile periods and independent variables has been assessed at the individual and community levels. With a p-value of less than 0.05, the strength was assessed and presented with an adjusted odds ratio (AOR) and 95% confidence intervals. Deviance = −2 (log likelihood ratio) was used to compare the models due to the nested nature of the model, and the model with the lowest deviance was chosen as the best-fit model. The variables used in the models were tested for multi-collinearity through assessing the variance inflation factors (VIF); the findings fell within the acceptable range of 1–10.

### 3.3 Ethical approval and consent to participate

This study is a secondary analysis of the DHS data, so it does not require ethical approval and consent to participate. For conducting our study, we registered and requested the dataset from DHS online archive and received approval to access and download the data files. According to the DHS report, all participant data were anonymized during the collection of the survey data. More details regarding DHS data and ethical standards are available online at http://www.dhsprogram.com.

## 4 Result

### 4.1 Socio-demographic and economic characteristics of adolescent girls and young women in sub-Saharan African countries

**Figure 1:**
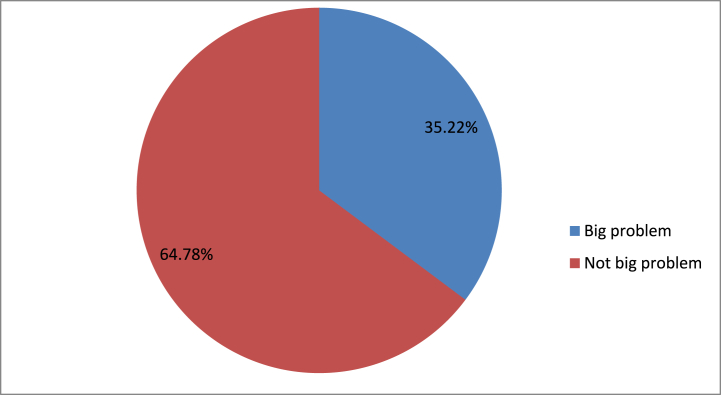
distance from health facility among adolecent girls and young wonem in sub-saharan africa

A total of 140,064 young women and adolescent girls in sub-Saharan Africa were involved in this study. Nineteen year was the study participants' median age (IQR: 17 to 22). Lesotho accounted for the smallest percentage of study participants (2842; 2.03%) while Nigeria accounted for the biggest percentage (15,267; 10.90%) of adolescent girls and young women in sub-Saharan Africa between the ages of 10 and 24. About 32.77% of participants were Catholic religious followers. Only 19.23% of adolescent girls and young women had internet access in Sub-Saharan African countries, and 14.18% of them heard about family planning. Nearly one-fifth of adolescent and young women (18.29%) did not attain formal education. Nearly two-thirds (61.86%) of the adolescent and young women were living in rural areas of sub-Saharan African countries. About more than half (58.44%) of study participants living in sub-Saharan African countries have poor community media exposure (Table 3). The study's participants perceive distance from the health facility to be a major issue, accounting for over one-third (35.22%) (Fig. 1).

**Table 3:** Socio-demographic and economic characteristics of adolescent and young women in Sub-Saharan Africa, DHS 2014–2020.

Variables	Frequency	Percent
Individual level variables		
Respondents age		
15-19	76,385	54.54
20-24	63,679	45.46
Respondents' educational status		
No education	25,616	18.29
Primary	47,080	33.61
Secondary and Higher	67,368	48.10
Currently working		
No	72,134	58.35
Yes	51,482	41.65
Religion		
Catholic	45,898	32.77
Muslim	1530	1.09
Orthodox	21,708	15.50
Protestant	6481	4.63
Others	64,447	46.01
Marital status		
Not married	83,668	59.74
Married	51,592	36.83
Ever married	4804	3.43
Husband education		
Not educated	16,742	33.47
Primary	14,236	28.46
Secondary and higher	19,050	38.08
Household wealth index		
Poor	53,052	37.88
Middle	27,443	19.59
Rich	59,569	42.53
Household media exposure		
No	81,858	58.44
Yes	58,206	41.56
Heard about family planning		
No	96,641	85.82
Yes	15,974	14.18
Use of internet		
No	93,309	80.77
Yes	22,218	19.23
**Community level variables**		
Residencer		
Urban	53,425	38.14
Rural	86,639	61.86
Community illiteracy		
Low	71,712	51.20
High	68,352	48.80
Community poverty		
Low	69,909	49.91
High	70,155	50.09
Community media exposure		
Low	73,854	52.73
High	66,210	47.27
Country category		
Southern SSA	9367	6.69
Western SSA	47,183	33.69
Eastern SSA	64,395	45.98
Central SSA	19,119	13.65

### 4.2 Magnitude of knowledge on fertile period among adolescent girls and young women in sub-Saharan Africa

**Figure 2:**
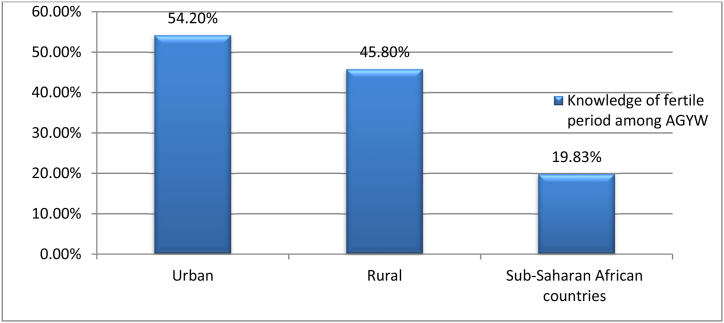
Magnitude of accurate konwledge on fertile period among adolescent girls and young women in sub-saharan africa countries.

**Figure 3:**
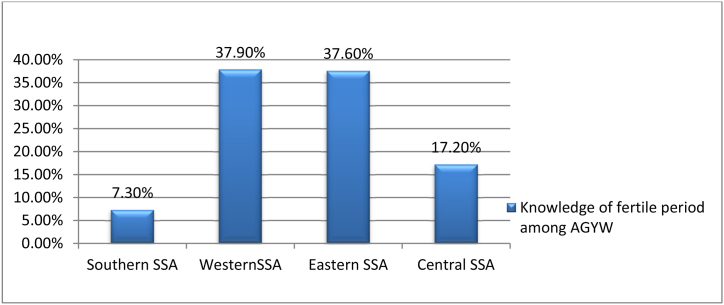
Regional of accurate konwledge on fertile period among adolescent girls and young women in sub-saharan africa countries.

In sub-Saharan Africa, adolescents girls and young women had a correct knowledge level of 19.83% (95% CI: 19.63, 20.04) regarding reproductive periods. Adolescent girls and young women in sub-Saharan African countries had accurate knowledge of fertile periods in 54.2% of urban and 45.8% of rural areas, respectively (Fig. 2). West Sub-Saharan African adolescents' girls and young women (37.9%) and Southern Sub-Saharan African adolescent's girls and young women (7.3%) had the highest and lowest accurate knowledge levels on fertile periods, respectively (Fig. 3).

### 4.3 Random effect and model fitness

A null model was used to assess if the data supported the decision to assess randomness at the community level. The results of the null model, which had a variance of 0.0820407 and a P value of 0.000, demonstrated that there were notable variations in the knowledge of fertile periods throughout cultures. 97.57% of the diversity in knowledge about fertile periods came from variations within clusters, whereas 2.43% of the difference came from variances across clusters. The likelihood of having accurate knowledge about fertile periods varied by a factor of 1.31 between the higher and lower clusters in the null model. According to Model I's intraclass correlation value, 2.02% of the variation in knowledge about the fertile period reflects the differences between communities. Next, we created Model II using community-level variables from Model I. Cluster variations were the basis for 2.15% of the differences in accurate knowledge of the fertile period, according to the ICC value from Model II. In the final model (model III), which attributed approximately 17.59% of the variation in the likelihood of accurate knowledge on fertile periods to both individual and community-level variables (Table 4), the likelihood of accurate knowledge varied by 1.28 times across inaccurate and accurate knowledge on fertile period clusters.

**Table 4:** Model comparison and random effect analysis for knowledge of fertile period among adolescent girls and young women in Sub-Saharan Africa.

Parameter	Null model	Model I	Model II	Model III
Random effect				
**Variance**	0.0820407	0.0676472	0.072288	0.0676129
**ICC**	2.43%	2.02%	2.15%	2.01%
**MOR**	1.31	1.28	1.29	1.28
**PCV**	Ref.	17.54%	11.89%	17.59%
**Model compression**				
**LLR**	−69506.945	−50353.108	−68719.44	−50115.375
**Deviance**	139,013.89	100,706.216	137,438.88‬	100,230.75

ICC: interacluster correlation, MOR: median odds ratio, PCV: proportional change in variance, LLR: logliklihood ratio.

### 4.4 Factors associated with the knowledge of fertile period among adolescent girls and young women in the sub-Saharan African countries

Age, educational status, proximity to a health facility, knowledge of family planning, community poverty, and place of residence were found to be strongly associated with respondents' knowledge of the fertile period among adolescent girls and young women in sub-Saharan African countries, according to the final fitted model of multivariable multilevel mixed effect logistic regression (Table 5).

**Table 5:** Multivariable multilevel logistic regression analysis of community and individual-level variables associated with adolescents' and young women's knowledge of the fertile period in Sub-Saharan Africa, DHS 2014–2020.

Variables	Model I	Model II	Model III
**Individual level factors**			
Respondents age			
15-19	1		1
20-24	1.41 (1.36, 1.46)		**1.44(1.40, 1.49)**
Educational status			
Not educated	1		1
Primary	0.86 (0.82, 0.90)		0.96 (0.91, 1.02)
Secondary and higher	1.60 (1.52, 1.68)		**1.68(1.60, 1.77)**
Currently working			
No	1		1
Yes	1.10 (0.07, 1.14)		1.09 (0.05, 1.13)
Religion			
Catholic	1		1
Muslim	1.36 (0.12, 1.64)		1.22 (0.01, 1.47)
Orthodox	1.02 (0.96, 1.08)		1.01 (0.96, 1.07)
Protestant	0.75 (0.69, 1.82)		0.70 (0.64, 1.76)
Others	1.10 (0.06, 1.13)		1.16 (0.12, 1.20)
Marital status			
Not married	1		1
Married	1.11 (1.07, 1.15)		1.10 (0.06, 1.14)
Ever married	1.01 (0.93, 1.11)		1.04 (0.95, 1.13)
Distance from heath facility			
Big problem	2.41 (2.36, 3.57)		
Not big problem	1		**2.31(2.02, 2.98)**
Wealth index			
Poor	1		1
Middle	1.09 (0.05, 1.15)		1.09 (0.04, 1.14)
Rich	1.26 (0.21, 1.32)		1.32 (0.26, 1.39)
Household media exposure			
No	1		1
Yes	0.99 (0.96, 1.03)		1.01 (0.97, 1.04)
Heard about family planning			
No	1		1
Yes	1.28 (1.22, 1.34)		**1.33(1.27, 1.39)**
Use of internet			
No	1		1
Yes	1.40 (1.34, 1.46)		1.35 (0.29, 1.40)
**Community level variables**			
Residence			
Urban		1.41 (1.37, 1.45)	**1.06(1.02, 1.10)**
Rural		1	1
Community illiteracy			
Low		1	1
High		1.00 (0.95, 1.04)	1.01 (0.96, 1.07)
Community poverty			
Low		3.62 (2.97, 3.87)	**3.06(3.01, 3.12)**
High		1	1
Community media exposure			
Low		1	1
High		0.97 (0.92, 1.02)	0.94 (0.89, 102)
Country category			
Southern SSA		1	1
Western SSA		1.03 (0.98, 1.10)	1.33 (0.21, 1.47)
Eastern SSA		0.73 (0.69, 0.77)	0.99 (0.89, 1.09)
Central SSA		1.17 (0.10, 1.24)	1.64 (0.48, 1.81)

SSA: Sub-Saharan Africa.

Adolescent girls and young women aged 20 to 24 had 1.44 times greater odds of accurately knowing the fertile period than did adolescent girls and young women aged 15 to 19 (AOR = 1.44, 95% CI: 1.40, 1.49). When compared to adolescent girls and young women with no formal education, knowledge of the fertile period was 1.68 times greater among those with secondary education or above (AOR = 1.68, 95% CI: 1.60, 1.77).

Adolescent girls and young women who had ever heard about family planning had 1.33 times greater odds of precise knowledge of the fertile period than did those who had not (AOR = 1.33, 95% CI: 1.27, 1.39). Adolescent girls and young women whose distance to a health facility is a major problem had 2.31 times higher odds of accurate knowledge about fertile periods than did those whose distance to a health facility is not a major problem (AOR = 2.31, 95% CI: 2.02, 2.98).

Adolescent girls and young women living in urban areas had 1.06 times higher odds of accurately knowing the fertile period than did those living in rural areas (AOR = 1.06, 95% CI: 1.02, 1.10). teenage girls and young women who lived in low community poverty levels had 3.06 times greater odds of accurate knowledge about fertile periods than did teenage girls and young women who lived in high community poverty (AOR = 3.06, 95% CI: 3.01, 3.12).

## 5 Discussion

Adolescent girls and young women's lives are significantly altered when they lack knowledge about fertility. Early motherhood restricts their ability to choose how to live the rest of their lives. Adolescent girls and young women's fertility also includes increased risks for the mother and baby's mortality and morbidity, as well as related challenges affecting their health. The development of adolescent girls and young women, as well as their capacity to attain high standards of health, education, and economic wellbeing, are impacted by very early childbearing. Moreover, the ability of adolescent girls and young women to enjoy their human and reproductive rights is further hampered by early childbearing, which frequently perpetuates a generational cycle of poverty, poor socioeconomic status, and gender inequality [25].

In this study, the magnitude of accurate knowledge on fertile periods among adolescent girls and young women in sub-Saharan African countries was found to be 19.83% (95% CI: 19.63, 20.04). This result is higher than studies carried out in Kenya, 8% [26], Ghana, 14.2% [27]. The possible reason for the discrepancies could be that, while the studies in Kenya and Ghana used primary data sources with smaller samples, our study, which was done at the level of sub-Saharan African countries, used secondary data from the DHS report. Furthermore, when compared to individual countries, certain sub-Saharan African countries have outstanding healthcare infrastructure and high-quality health services for adolescent girls and young women.

On the other hand, accurate knowledge regarding the fertile period among the young women and adolescent girls in this study was lower than the results obtained in Baghdad, 36% [28] and United States, 29% [29]. The lower knowledge on fertile periods among adolescent girls and young women in this study could be due to adolescent and young women's education systems regarding fertility, the quality of adolescent and young women's healthcare services, and the fact that economic and health policies in Baghdad and the United States are better compared with those of Sub-Saharan African countries. Moreover, adolescent and young women's attitudes towards health service utilization and differences in religious and cultural practices across countries could be other causes of variability in knowledge level during the fertile period.

It was discovered that knowledge of the fertile period among AGYW was associated with both individual and community-level variables in the final fitted model of multivariable multilevel logistic regression. Age, educational attainment, distance to a health facility, awareness with family planning, level of poverty in the community, and residence were all strongly associated with the respondents' knowledge of the fertile period among young women and adolescent girls in sub-Saharan African countries.

In this study, adolescent girls and young women between the ages of 20 and 24 had 1.44 times higher odds of accurate knowledge of the fertile period than their younger counterparts (15–19 years). This result is in line with earlier findings in Refs. [27,30]. The possible explanation for the association might be that adolescent girls and young women in the lower age extremities have less knowledge about the fertility period than the older ones, possibly because of shorter sexual experiences among the younger adolescent girls and women or because older adolescent girls and young women are more informed through sex education than the younger ones. Adolescent girls and young women's sexual and reproductive knowledge is improved as they become older and are exposed to more reproductive-related issues [31].

On the other hand, the higher age group might give them more chances to receive a lot of pertinent information because the higher age group is more likely to be employed, while studies showed that being employed was found to increase the chance of having good knowledge about fertility periods. This might be explained as the information needed about fertility periods can be easily obtained from their colleagues. In addition, employed adolescent girls and young women could improve the use of health information, like the advantages of understanding fertility periods.

Knowledge of the fertile period among adolescent girls and young women was found to be significantly associated with educational level. Comparing adolescent girls and young women with secondary education or above to those without, they found that the former had 1.68 times greater knowledge of the fertile period. This study's finding is supported by the previous findings [27,[32], [33], [34]]. This may be due to the fact that adolescent girls and young women with higher educational levels have probably received some kind of sex education that covers topics like when to get pregnant and other female reproductive concerns; therefore, higher education indicates greater fertility knowledge [35].

Furthermore, a plausible explanation could be that adolescent girls and young women with higher levels of education are inclined to seek medical attention from a qualified practitioner. Higher educated young women and adolescent girls typically comprehend pregnancy-related problems and have a better understanding of self-care. Possess more authority when making decisions for the family and are better informed about the advantages of suggested family planning options as well as pregnancy-related complications.

Adolescent girls and young women where distance to a health facility is a major problem had 2.31 times greater odds of accurately knowing the fertile period than did similar groups where getting to a health facility is not a major concern. This is corroborated by earlier research done in Ref. [36]. The reason for this could be that if the distance to a health facility is not considered a barrier, adolescent girls and young women are more likely to utilize health services such as family planning due to easy access to transportation services to the health facility, and consequently, they would acquire good knowledge of the fertile period.

Adolescent girls and young women who had ever heard about family planning messages from the media had 1.33 times higher odds of having precise knowledge about the fertile period than did those who had not. This is in line with the research done in Ref. [37]. The plausible explanation is that adolescents and young women who received family planning messages were more likely to be well-informed about the fertile period. Individuals who were exposed to this kind of media exposure are therefore more likely than those who were not to have a solid knowledge about the fertile period.

Compared to adolescent girls and young women living in rural areas, the odds of accurately knowing the fertile period were 1.06 times greater for those living in urban residents. This is in line with the previous studies conducted in Refs. [34,38,39]. The possible explanation might be that those adolescent girls and young women living in urban areas of Sub-Saharan Africa countries have increased access to media, the internet, and websites, good financial capability to afford transportation costs to get adolescent health services in the health facility, good awareness of utilizing health facilities, and better availability of nearby health care services, which leads them to have good knowledge on fertile periods.

Furthermore, this is due to the fact that adolescent girls and young women who live in rural areas are less likely to utilize maternal health services like family planning methods because of their inadequate availability and accessibility and due to the unequal distribution of health facilities as well as health personnel between urban and rural areas.

Adolescent girls and young women living in economically privileged communities had 3.06 times higher accurate knowledge of the fertile period compared to adolescent girls and young women living in lowest community wealth quintile. This finding is in line with other studies in Refs. [31,33]. This association might be a result of the fact that good knowledge among adolescent girls and young women with advanced community economic status increases their desire to know about fertility issues [40]. Moreover, this might be due to the higher chance of getting adequate knowledge about fertility periods among that higher wealth status than poor wealth status as they may not be afford to buy media sources such as television, radio, and newspapers which are expensive for people with low income.

Using the most recent big representative sample size of 140,064 and using the proper statistical analysis (mixed effect multilevel analysis) was one of the study's main strengths. It measures adolescent and young women's knowledge of the fertile period and determinant factors at individual and community levels in 23 sub-Saharan African countries and is believed to be generalizable to other developing countries. However, certain sub-Saharan African countries have not conducted a demographic and health survey since 2014, which could have affected the representativeness of our findings. Another limitation of our study was the absence of some important variables in DHS dataset such as social media usage which may have a greater impact on knowledge of fertile periods.

## 6 Conclusions and recommendation

In this study, the magnitude of accurate knowledge about fertile periods among adolescent girls and young women in sub-Saharan African countries was found to be 19.83%. The study identified that both individual-level (respondents’ age, educational status, distance to a health facility, knowledge of family planning) and community-level (community poverty, and place of residence) variables were determinants of knowledge about the fertile period. Therefore, the governments and ministries of health in sub-Saharan African countries should give attention to those adolescent girls and young women who reported distance as a big problem in accessing reproductive health services and to rural resident adolescent girls and young women while designing policies and strategies targeting improving awareness on fertility issues in sub-Saharan Africa.

## Consent for publication

Not applicable.

## Data availability statement

Data will be made available upon request.

## Funding statement

There are no funders to report for this submission.

## CRediT authorship contribution statement

**Alebachew Ferede Zegeye:** Validation, Supervision, Software, Resources, Project administration, Methodology, Investigation, Funding acquisition, Formal analysis, Data curation, Conceptualization. **Tadesse Tarik Tamir:** Resources, Project administration, Methodology, Investigation, Funding acquisition, Data curation. **Belayneh Shetie Workneh:** Writing – review & editing, Validation, Supervision, Software, Project administration, Formal analysis. **Wubshet Debebe Negash:** Visualization, Supervision, Formal analysis, Data curation, Conceptualization. **Chilot Kassa Mekonnen:** Supervision, Software, Resources, Project administration, Conceptualization, Writing – original draft, Validation, Software, Methodology, Investigation, Funding acquisition, Formal analysis, Data curation, Conceptualization.

## Declaration of competing interest

The authors declare that they have no known competing financial interests or personal relationships that could have appeared to influence the work reported in this paper.
